# Challenges in Double-Lumen Tube Placement Due to Idiopathic Laryngotracheal Stenosis in a Right Lung Adenocarcinoma Patient: A Case Report

**DOI:** 10.7759/cureus.78366

**Published:** 2025-02-01

**Authors:** Marie Fujii, Daisuke Sugiyama, Eri Matsuura, Kenichi Ueda

**Affiliations:** 1 Department of Anesthesiology, Kameda Medical Center, Kamogawa, JPN; 2 Department of Anesthesiology, University of Iowa Hospitals and Clinics, Iowa City, USA

**Keywords:** bronchial blocker, double-lumen tube, idiopathic laryngotracheal stenosis, one-lung ventilation, upper tracheal stenosis

## Abstract

We report a case of idiopathic laryngotracheal stenosis (ILTS) in a 72-year-old woman scheduled for a right upper lobectomy. Although she had no history of tracheal intubation or respiratory disease, her bronchus was stenosed for approximately 2 cm starting 5 cm below the vocal cords, with the narrowest lumen measuring 10 mm, which impeded placement of a 32 Fr double-lumen tube (DLT). Consequently, a 7.5 mm endotracheal tube with a bronchial blocker was used for lung isolation. Even without a history of tracheal stenosis, it is crucial to evaluate potential stenosis using CT or endoscopy. If symptoms occur, endoscopic or surgical treatment should be considered.

## Introduction

Tracheal stenosis can arise from various causes, including trauma, inflammation, and burns. Idiopathic laryngotracheal stenosis (ILTS) is identified when the underlying cause is unknown. ILTS is characterized by lesions that originate at the cricoid cartilage and extend from the subglottic region to the first tracheal ring [1]. The most severe cases typically occur from the lower part of the glottis to the first tracheal ring [1]. In this instance, however, we encountered a case where the stenosis was located at an unconventional site, deviating from the typical ITLS presentation. We report a case in which the placement of a double-lumen tube (DLT) for one-lung ventilation (OLV) was not achieved due to tracheal stenosis at an atypical site, complicating secure airway and management.

## Case presentation

A 72-year-old woman, with the American Society of Anesthesiologists (ASA) physical status Ⅱ, 152 cm in height and 37 kg in weight, with right upper lobe lung adenocarcinoma was scheduled for thoracoscopic right upper lung resection under general anesthesia and epidural anesthesia. She had hypertension, gastric ulcer, mild cognitive impairment, and no relevant surgical history. After placing the epidural catheter, general anesthesia was induced with intravenous fentanyl (100 μg), propofol (40 mg), remifentanil (0.2 μg/kg/min infusion), and rocuronium (40 mg). First, after confirming that the train of four (TOF) count was 0, we tried to intubate the lubricated DLT (35 Fr in outside diameter; Broncho-Cath^TM^ Left, Covidien, Mansfield, USA) with video laryngoscope (McGRATH^TM^ MAC^TM^, Covidien) to achieve OLV. Although visualization of the epiglottis and vocal cords was obtained without difficulty, there was resistance as we passed the DLT through the vocal cords. Consequently, a flexible bronchoscope was threaded through the 35 Fr DLT to investigate the anatomy. Bronchoscopic examination revealed a tracheal narrowing a few centimeters distal to the glottis, which prevented the passage of the tube. An attempt was made to intubate the DLT with a smaller size (32 Fr) without success. Subsequently, the 32 Fr DLT was removed and intubation was successfully performed with a standard endotracheal tube (7.5 mm in internal diameter; Shiley^TM^ Endotracheal Tube with TaperGuard^TM^ Cuff, Murphy Eye with optional Preloaded Stylet, Covidien). A bronchial blocker (3.0 mm in outside diameter; COOPDECH^TM^ bronchial blocker tube type A; Daiken Medical, Osaka, Japan) was then placed through the tracheal tube to establish OLV. The patient was then placed in the left lateral decubitus position and surgery was started. Anesthesia was maintained with 4% desflurane, with pure oxygen until OLV was established, after which the oxygen concentration was titrated at 50%. OLV was maintained effectively throughout the operation without any problems, and the surgery was successfully completed. After extubation, the patient was transferred to the high-care unit with a 5 L oxygen mask in place. The patient was discharged from the hospital on postoperative day seven.

## Discussion

ILTS is usually observed in Caucasian women with an average age of 50 years [2,3]. This condition is notably rare, with an incidence of 1:400,000 annually in the United States [4]. Patients typically present with dyspnea on exertion that progresses to dyspnea at rest or stridor with symptoms developing over the course of months to years [1]. Recent studies have shown that subglottic stenosis in ILTS patients involves the upregulation of fibroblasts and interleukin-17A (IL-17A), leading to pathological fibrosis [5]. Since the majority of patients are female, there has been speculation regarding a hormonal link, specifically to estrogen. While recent studies have explored estrogen’s potential role in pathogenesis, the definitive etiology remains unclear [5]. Furthermore, there is a possible association between ILTS and gastroesophageal reflux disease, indicating that inflammation from gastric acid reflux may contribute to the development of this condition [6-8].

In the management of ILTS, preoperative evaluation and tailored interventions based on the severity of the stenosis are essential. To begin with, it is crucial to confirm the upper and lower boundaries of the stenosis as well as the diameter of the stenotic lumen using CT or endoscopic examination [1]. Additionally, attention should be paid to the presence of lesions in other areas, whether the stenosis is fresh initial granulation or mature cicatricial stenosis, and to any atypical features or nasal abnormalities associated with autoimmune or infectious etiologies. If these are observed, a biopsy may be warranted [9]. Generally, after assessing the location, length, and lumen diameter of the stenosis, an appropriate tracheal tube should be selected. If there are concerns about intubation after anesthesia induction, awake fiberoptic intubation or a tracheostomy may be necessary. Depending on the degree of stenosis and symptoms, endoscopic or surgical treatment may be performed, although surgery is rarely required. Tracheostomy is performed to secure the airway in cases of acute onset dyspnea or before surgical intervention, though cases actually requiring tracheostomy are uncommon [9]. Options to dilate the stenotic area include balloon dilation, laser ablation, and stent placement.

**Figure 1:**
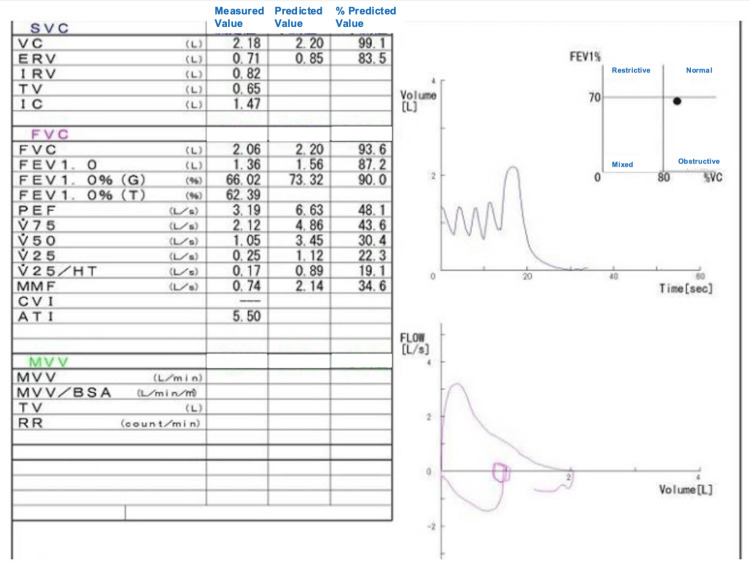
Preoperative respiratory function test results. The preoperative respiratory function test results were mostly within the normal range, and no severe obstructive impairment was observed. SVC: slow vital capacity; VC: vital capacity; ERV: expiratory reserve volume; IRV: inspiratory reserve volume; IC: inspiratory capacity; FVC: forced vital capacity; FEV: forced expiratory volume; PEF: peak expiratory flow; HT: height; MMF: maximal mid-expiratory flow; CV: closing volume; AT: anaerobic threshold; MVV: maximum voluntary ventilation; BSA: body surface area; TV: tidal volume; RR: respiratory rate.

**Figure 2:**
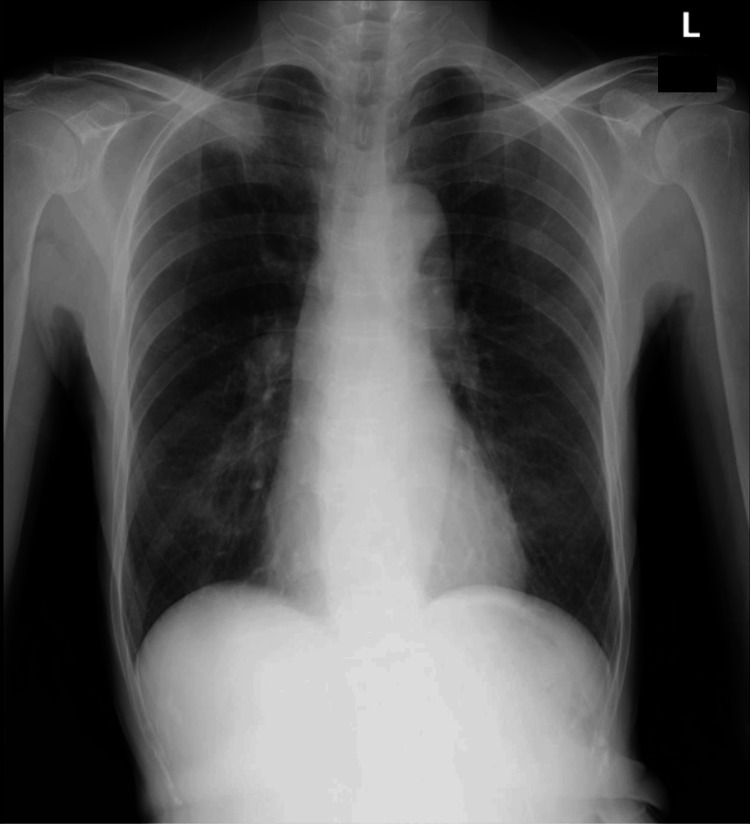
Results of preoperative chest X-ray. The preoperative chest X-ray findings did not clearly indicate tracheal stenosis.

In the present case, preoperative pulmonary function test results were almost within the normal range (Figure 1), and the preoperative chest X-ray did not clearly indicate stenosis (Figure 2), making it difficult to predict ILTS before surgery.

**Figure 3:**
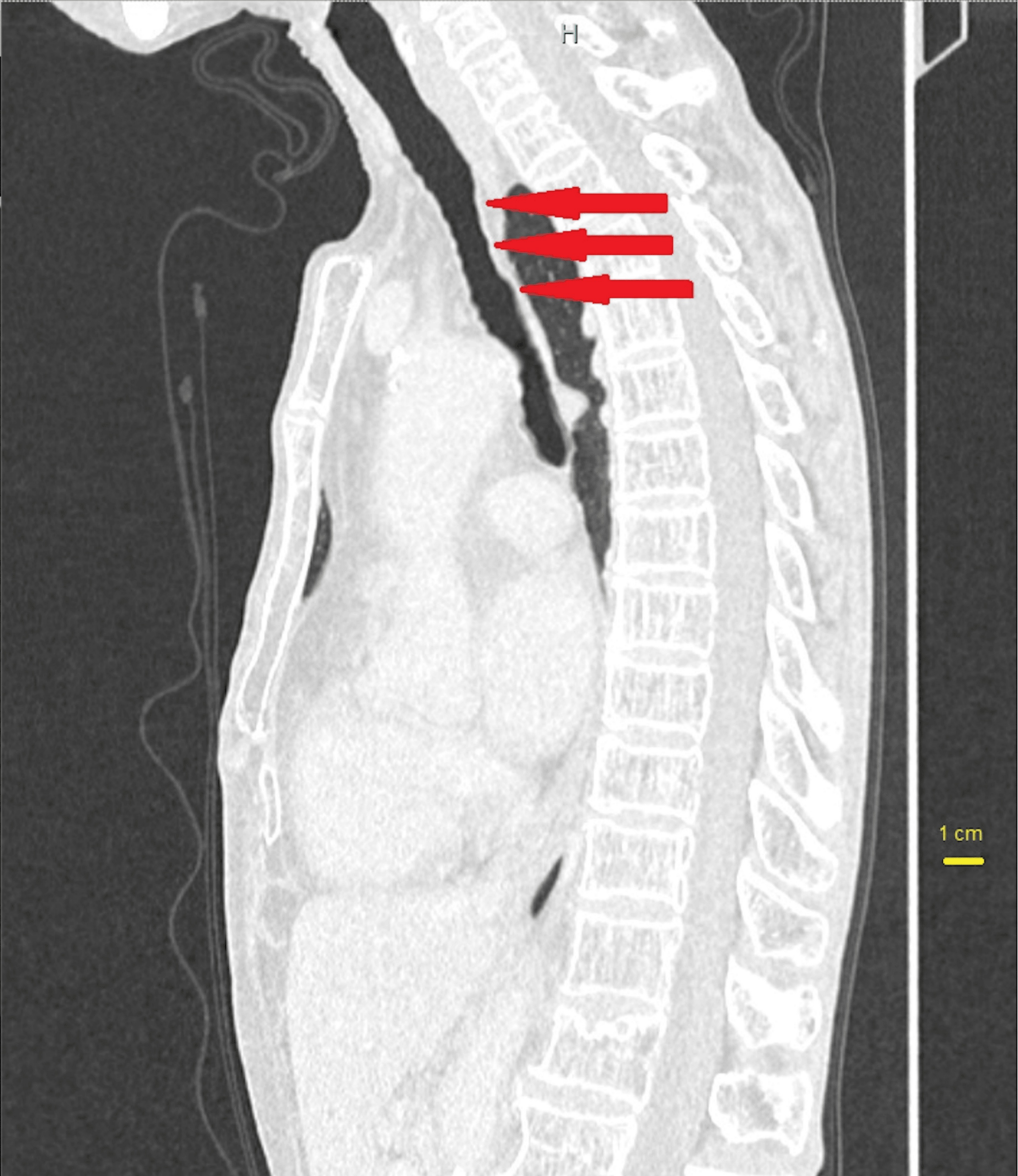
Chest CT image before surgery in sagittal section. Red arrows indicate tracheal stenosis. CT revealed the stenosis is located 5 cm distal to the glottis and the narrowest inner diameter of the stenosis is approximately 10 mm.

**Figure 4:**
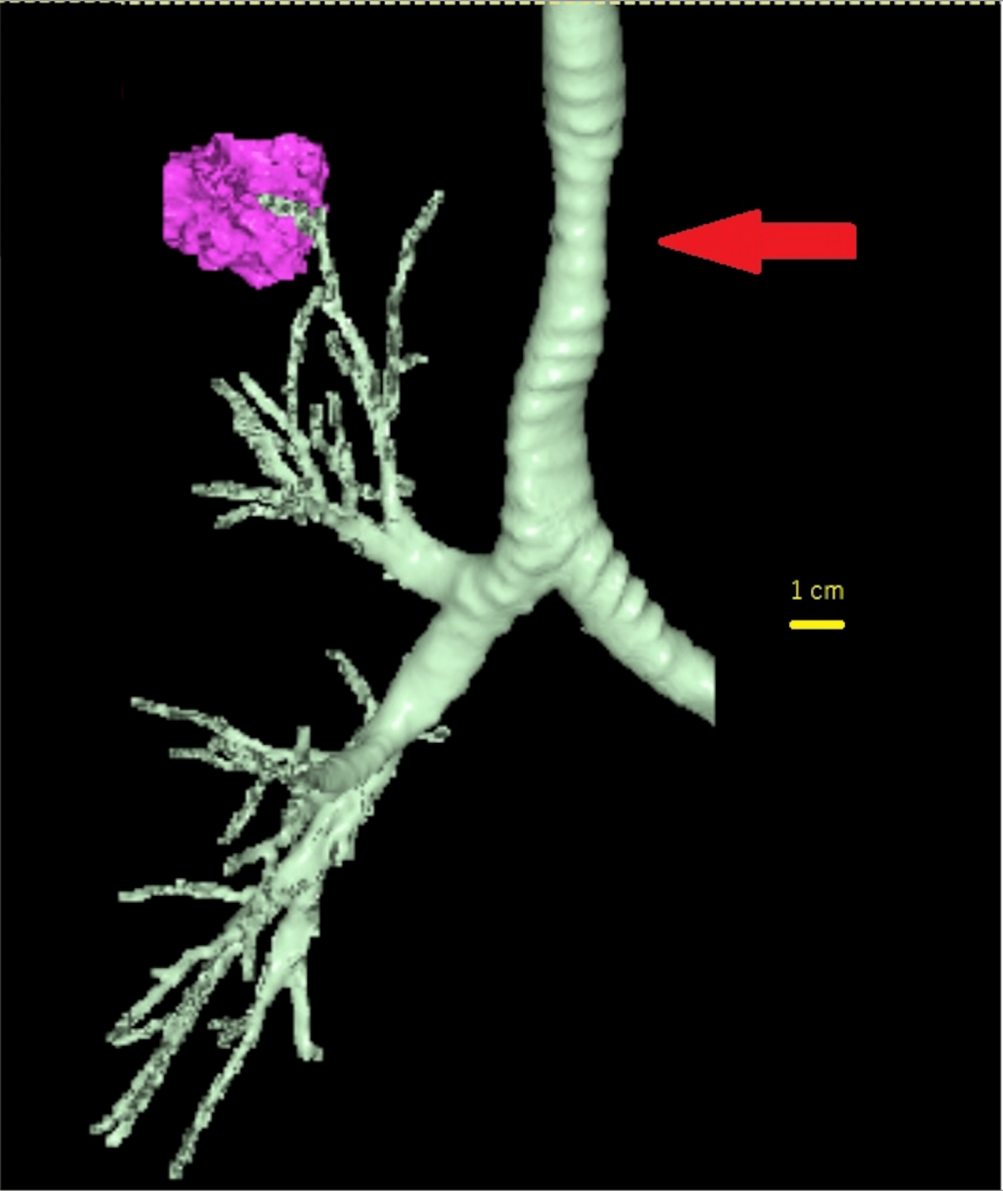
3D image constructed from a chest CT scan. The red arrow indicates the tracheal stenosis site, which spans approximately 2 cm in the cephalocaudal direction.

Additionally, there were no proceeding events or medical history suggestive of tracheal stenosis, leading to the consideration of ILTS. The tracheal stenosis in this patient commenced 5 cm distal to the glottis, with the constricted segment extending approximately 2 cm in the cephalocaudal direction. While the length of the stenosis aligns with typical presentations, its location is more peripheral than commonly affected areas (Figures 3, 4). Furthermore, the patient was Japanese, a demographic for which no prevalence has been reported, making this case particularly unusual.

In this case, a 7.5 mm endotracheal tube alongside a 9 Fr bronchial blocker was used to establish isolated pulmonary ventilation for the tracheal stenosis at the point of maximal narrowing approximately 10 cm in length (Figure 1). Given the rarity of ILTS, meticulous preoperative evaluation of the airway’s site and the degree of stenosis is essential, utilizing both chest X-ray and CT imaging, with CT providing superior diagnostic accuracy. Simulation of tailored airway management strategies is paramount. If the patient exhibits increased dyspnea and stridor, and if the stenosis is severe, awake intubation using a fiberoptic bronchoscopy should be considered. In cases where a 7.5 mm endotracheal tube, which is the smallest size recommended by the manufacturer for use with a 9 Fr bronchial blocker, cannot be placed, a 7 Fr or smaller bronchial blocker is an option [10]. Alternatively, a bronchial blocker can be placed ex-situ of the endotracheal tube. Non-urgent cases may warrant preoperative endoscopic or surgical intervention in consultation with a surgeon.

## Conclusions

In conclusion, ILTS does not necessarily occur in typical races or typical locations. Therefore, thorough airway evaluation and airway management planning should be conducted over a wide range based on physical findings and imaging studies such as CT and bronchoscopy. For cases where airway management is expected to be difficult, awake intubation or tracheostomy should be considered. Balloon dilation, laser ablation, and tracheal stenting should also be considered when necessary.
